# The Importance of Accurate Drug Use History in Diagnosing and Managing Cannabinoid Hyperemesis Syndrome: A Case Report

**DOI:** 10.7759/cureus.80352

**Published:** 2025-03-10

**Authors:** Sara Filipa Silva, Maria Beatriz Couto

**Affiliations:** 1 Fafe Sentinel Family Health Unit (Unidade de Saúde Familiar Fafe Sentinela), Unidade Local de Saúde do Alto Ave, Guimarães, PRT; 2 Department of Psychiatry, Unidade Local de Saúde do Alto Ave, Guimarães, PRT

**Keywords:** abdominal pain, cannabinoid hyperemesis syndrome, cannabinoids, cannabis use, cyclic vomiting, hot baths, marijuana abuse

## Abstract

Cannabinoid hyperemesis syndrome (CHS) is a paradoxical condition characterized by recurrent episodes of nausea, vomiting, and abdominal pain in chronic cannabis users. Despite the increasing legalization and widespread use of cannabis, CHS remains underdiagnosed, often leading to unnecessary diagnostic tests and ineffective treatments. The syndrome is marked by symptom relief through hot baths, a behavioral pattern that can aid in its identification.We report a case of a 29-year-old male with a five-year history of recurrent vomiting and significant weight loss. The patient had multiple emergency department visits and was misdiagnosed with anxiety disorder due to the absence of significant findings on diagnostic tests. His condition was further complicated by a concurrent *Helicobacter pylori *infection, which delayed the clinical suspicion of CHS. A detailed assessment revealed a prolonged history of daily cannabis use and symptom relief through compulsive hot bathing. Upon cessation of cannabis use and symptomatic treatment with mirtazapine, quetiapine, and lorazepam, the patient showed complete resolution of symptoms and remained asymptomatic.This case highlights the diagnostic challenges of CHS and underscores the importance of targeted questioning about cannabis use in patients presenting with cyclical vomiting. Clinicians should maintain a high index of suspicion, especially in cases resistant to conventional antiemetic therapy. Early recognition and patient education regarding cannabis cessation are critical to preventing recurrence and improving long-term outcomes.

## Introduction

Cannabinoid hyperemesis syndrome (CHS) is an underrecognized condition characterized by recurrent episodes of nausea, vomiting, and abdominal pain in individuals with chronic cannabis use [1]. First described in 2004, CHS has gained attention in recent years due to the increasing prevalence of cannabis consumption, especially following legalization in various regions [2]. Despite this, the syndrome remains frequently misdiagnosed, often mistaken for cyclic vomiting syndrome, gastrointestinal disorders, or psychiatric conditions, leading to unnecessary diagnostic tests and ineffective treatments.

A key feature of CHS is the paradoxical effect of cannabis, a substance widely known for its antiemetic properties, which, in chronic users, can instead induce persistent nausea and vomiting [3]. A distinctive characteristic of CHS is the compulsive use of hot showers or baths as a temporary means of symptom relief, a behavioral marker that can assist in its clinical identification [4]. However, due to the stigma surrounding cannabis use and the reluctance of patients to disclose their substance habits, obtaining an accurate history can be challenging [5].

This report presents the case of a 29-year-old male with a prolonged history of recurrent vomiting and epigastric pain, leading to multiple emergency department visits and consultations across various specialties. His condition was initially attributed to anxiety disorder, resulting in delayed diagnosis and prolonged suffering. Through this case, we emphasize the importance of recognizing CHS, incorporating targeted questioning about cannabis use, and considering the syndrome in the differential diagnosis of recurrent vomiting. Early identification and patient education on cannabis cessation are crucial to prevent recurrence and improve outcomes.

## Case presentation

A 29-year-old male patient, single and childless, working as a freelancer, presented with no significant medical history, no regular medication use, and no relevant family history. Regarding substance use, he reported cannabis consumption since the age of 19 but was currently abstinent. He denied the use of other drugs and alcohol; however, he was an active smoker, consuming approximately one pack (20 cigarettes) per day.

**Figure 1:**
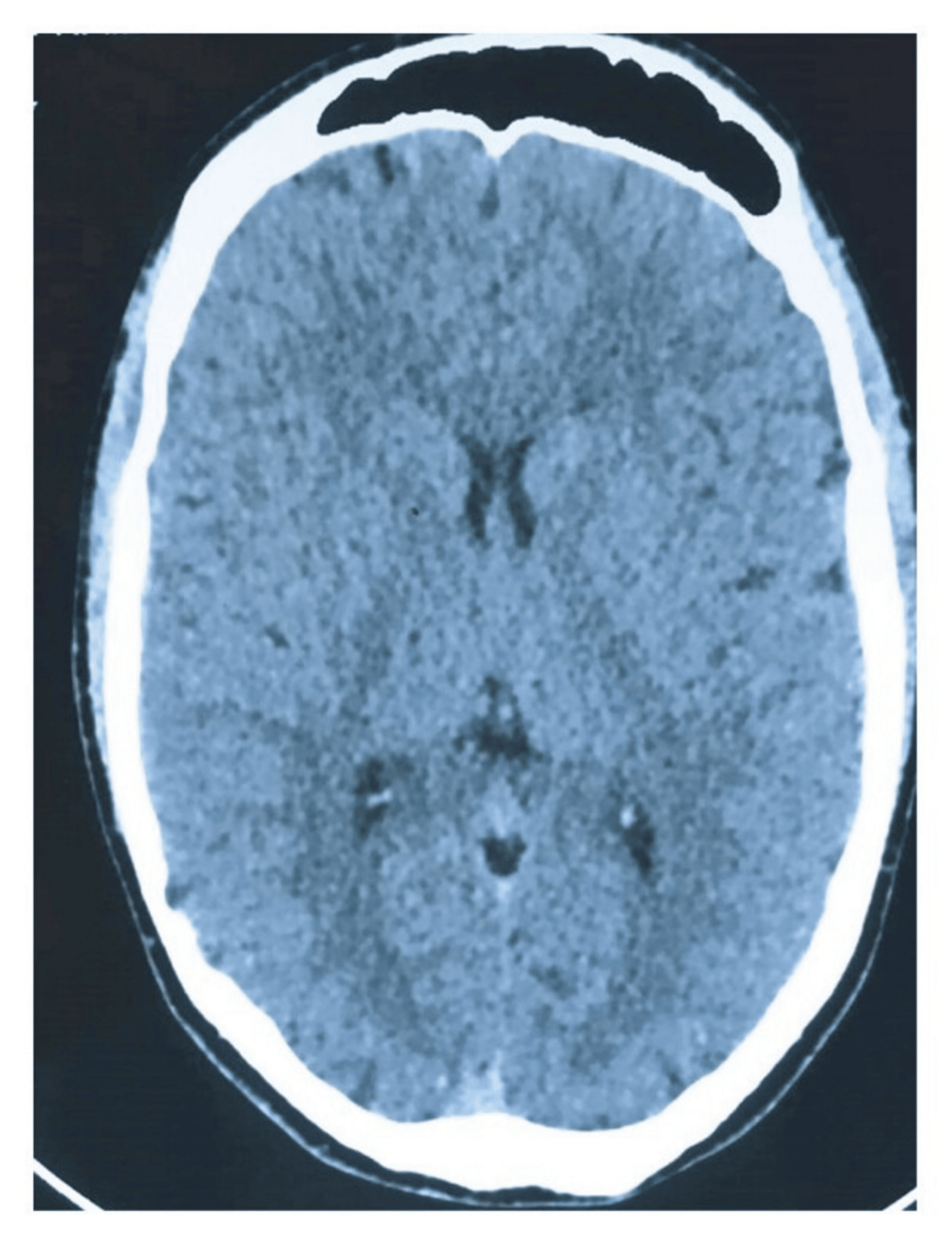
Cerbral CT scan shows normal results

The patient had a history of recurrent nausea and vomiting occurring approximately once every three to four weeks, with episodes lasting two to three days, associated with epigastric abdominal pain, loss of appetite, and food intolerance for five years. Initially, these episodes were mild and sporadic but gradually became more frequent and severe over time. During this period, he had multiple consultations in primary healthcare and emergency departments and was even evaluated by general surgery. However, blood tests and imaging studies always showed no significant alterations (Figure 1). A CT scan performed during one of these visits was normal, effectively ruling out structural causes of the symptoms and increasing suspicion of a functional or substance-related disorder. Symptomatic treatment was provided. He was repeatedly diagnosed with anxiety disorder.

He stated: *"I didn't feel anxious, nor did I have any reason to, but everyone kept telling me this, and I ended up becoming anxious about the situation itself."* He was prescribed anxiety medication but did not experience any improvement.

In July 2024, he returned to the emergency department with the same symptoms: experiencing uncontrollable vomiting episodes for the past seven days, associated with epigastric pain (7/10 in intensity), without radiation. No fever, no gastrointestinal transit changes, no history of sick contacts, and no abnormalities were identified in diagnostic tests (Table 1). Laboratory results showed mild electrolyte imbalance, with low potassium (K+ 3.2 mmol/L), which is commonly seen in patients with prolonged vomiting. However, no signs of systemic infection or other major abnormalities were noted. This time, a psychiatric consultation was requested.

**Table 1:** Laboratory results BUN: blood urea nitrogen; WBC: white blood cells; M: male; F: female

Serum Tests	Patient Values	Reference Ranges
Glucose	174 mg/dl	90-170 mg/dl (Randon)
Sodium	138 mEq/l	135-145 mEq/l
Potassium	3.3 mEq/l	3.5-5.0 mEq/l
Chloride	108 mEq/l	96-106 mEq/l
Bicarbonate	26 mEq/l	22-28 mEq/l
BUN	14 mg/dl	7-20 mg/dl
Creatine	0.9 mg/dl	0.6-1.3 mg/dl
Hemoglobin	13.7 g/dl	13.5-17.5 g/dl (M), 12-15.5 g/dl (F)
Hematocrit	39 %	38-50% (M), 34-44% (F)
WBC	9.4 × 10⁹/liter	4.0-11.0 × 10⁹/liter
Platelets	259 × 10⁹/liter	150-450 × 10⁹/liter

During this evaluation, his daily cannabis use since the age of 19 (approximately 2 g/day) and active smoking (20 cigarettes/day) were identified. He denied the use of other drugs. Notably, the patient's symptoms had started shortly after initiating daily cannabis use and had progressively worsened over time, correlating with his continued cannabis consumption. He reported a weight loss of 30 kg (body mass index (BMI) decreased from 24.6 in 2019 to 16.3 in 2024) since symptom onset five years ago. He identified hot baths as the only relief factor:* "I felt fine under the water, but as soon as I stepped out, I immediately felt bad again."*

He was then diagnosed with CHS and started on mirtazapine, quetiapine, and lorazepam. Mirtazapine was selected for its appetite-stimulating and antiemetic properties, helping to counteract the patient's significant weight loss and nausea. Quetiapine was prescribed to manage associated anxiety and sleep disturbances, while lorazepam was used for its anxiolytic and sedative effects, aiding in symptom relief and overall stabilization. The patient continued this medication regimen until September 2024, with gradual tapering.

A critical aspect of his treatment was complete cannabis cessation, as ongoing cannabis use is known to perpetuate CHS symptoms. Abstinence is the only definitive intervention that leads to sustained symptom resolution, as continued exposure to cannabinoids directly triggers and maintains the hyperemetic episodes. Since diagnosis, the patient has remained abstinent and has not reported any recurrence of symptoms. Currently, he has no depressive or anxiety symptoms, no other complaints, and maintains normal sleep patterns.

From 2019 to the present, the patient underwent multiple diagnostic tests, including two endoscopic studies identifying *Helicobacter pylori* and four eradication attempts. The overlapping symptoms of Helicobacter pylori infection and CHS, combined with the intermittent nature of the vomiting episodes, contributed to the delayed diagnosis. The failure to recognize the temporal relationship between cannabis use and symptom exacerbation further complicated the diagnostic process, leading to repeated misdiagnoses. Due to the similarity of symptoms, this may have directly contributed to the delayed diagnosis of CHS.

## Discussion

The diagnosis of CHS depends on complete medical history reports and detailed physical exams that exclude all possible reasons for hyperemesis [5]. Basic blood tests together with renal function assessments and urine drug screening (for cannabinoid detection) and required imaging studies should initiate the diagnostic evaluation [6]. The cycling pattern of CHS leads healthcare providers to mistake the disorder with cyclic vomiting syndrome and psychogenic vomiting but its distinguishing features are long-term cannabis use and response to hot baths and repeated nausea and vomiting episodes. The diagnostic challenges of CHS become more complicated due to the minimal success rates of standard anti-emetic medications metoclopramide and ondansetron [7].

Healthcare professionals are working to increase CHS awareness while facing the absence of established procedures for emergency room diagnosis and treatment of this condition. Medical resources get strained because CHS patients must take various diagnostic tests and spend additional time in emergency departments and hospitals before receiving a correct diagnosis [8]. CHS's long-term recurring nature increases healthcare system strain thus requiring better cannabis complication diagnostic methods and public awareness about marijuana-associated health problems.

Patient denial to report cannabis consumption is a principal obstacle in early CHS diagnosis mainly because individuals view cannabis as harmless and regard it as an antiemetic agent. The delayed revelation of cannabis use results in clinical suspicions being delayed as well as the need for proper interventions. Hot showering behavior serves as an effective screening tool for CHS since more than 90% of CHS patients exhibit this symptom without requiring specific drug-use questions [9]. Urine drug screenings must be used when historical examination fails to provide clear evidence regarding the recent use of cannabinoids.

The global cannabis legalization movement predicts that CHS cases will grow while emergency department encounters and hospital admissions increase because of complications from cannabis use. The exact way CHS develops remains elusive to scientists who need to conduct additional research to clarify cannabis-related nausea and vomiting processes [10]. Medical organizations should understand CHS risk factors because cannabis, which is often prescribed for chemotherapy nausea and pain relief, causes unexpected vomiting in chronic users [11].

Disease diagnosis through screening is vital because urine drug tests and specific medical inquiries work as the primary detection tools. Healthcare professionals must provide detailed information about the diagnosis of the syndrome to patients followed by clear instructions to stop marijuana use to prevent future occurrences. As the number of CHS cases is expected to increase, healthcare workers need to actively teach public audiences about cannabis dangers while stressing that using cannabis carries risks [12].

Since its original description as CHS in 2004, there have been increased publications on the syndrome in the literature; however, patients and health professionals still lack proper understanding, thus creating a need for continuous education efforts accompanied by the development of standard management guidelines [13].

## Conclusions

CHS remains an underrecognized condition, often leading to delayed diagnosis and unnecessary medical investigations. This case highlights the importance of obtaining a detailed substance use history and recognizing key clinical features such as cyclic vomiting, abdominal pain, and symptomatic relief with hot showers. However, a major challenge in diagnosing CHS lies in patients’ reluctance to disclose cannabis use, which can be a significant barrier to timely treatment. Given the increasing prevalence of cannabis use, healthcare professionals must maintain a high index of suspicion for CHS, especially in patients unresponsive to conventional antiemetics. Clinicians should ask targeted questions about cannabis use, particularly when treating patients presenting with unexplained cyclical vomiting or abdominal pain, as early identification and patient education are crucial for successful management. Cannabis cessation remains the cornerstone of treatment, preventing recurrence and reducing the healthcare burden.
